# Macrogol (polyethylene glycol) laxatives in children with functional constipation and faecal impaction: a systematic review

**DOI:** 10.1136/adc.2007.128769

**Published:** 2008-11-19

**Authors:** D Candy, J Belsey

**Affiliations:** 1 Paediatric Gastroenterology Service, Royal West Sussex NHS Trust, Chichester, UK; 2 JB Medical Ltd, The Old Brickworks, Chapel Lane, Little Cornard, Sudbury, UK

## Abstract

As the evidence base supporting the use of laxatives in children is very limited, we undertook an updated systematic review to clarify the issue. A comprehensive literature search was carried out to identify randomised controlled trials of polyethylene glycol (PEG) versus either placebo or active comparator, in patients aged <18 years with primary chronic constipation. Outcomes were assessed as either global assessments of effectiveness or differences in defaecation rates. Seven qualifying studies involving 594 children were identified. Five were comparisons of PEG with lactulose, one with milk of magnesia and one with placebo. Study duration ranged from 2 weeks to 12 months. PEG was significantly more effective than placebo and either equivalent to (two studies) or superior to (four studies) active comparator. Differences in study design precluded meaningful meta-analysis. Lack of high quality studies has meant that the management of childhood constipation has tended to rely on anecdote and empirical treatment choice. Recent publication of well designed randomised trials now permits a more evidence-based approach, with PEG-based treatments having been proven to be effective and well-tolerated first-line treatment.

**What is already known on this topic**

- Current treatment of childhood constipation tends to focus on oral rather than rectal treatments.
- Agents such as lactulose and senna are still widely used despite the lack of any meaningful clinical trial evidence.
- Since the last systematic review was published, several new randomised controlled trials have suggested that polyethylene glycol-based laxatives are effective in this patient group.

**What this study adds**

- Children with constipation treated with polyethylene glycol (PEG)-based laxatives have demonstrated consistently good outcomes.
- The efficacy of PEG is as good as or better than lactulose or milk of magnesia over a wide range of ages and treatment durations.
- PEG has the added advantage of being an effective disimpacting agent.

The last decade has seen a transformation in the management of constipation in children. The emphasis has changed from disimpacting the colon with suppositories and enemas or manual removal followed by polypharmacy with osmotic and stimulant laxatives. Although individual practice varies, nowadays there is widespread use of macrogol (polyethylene glycol, PEG)-based laxatives to empty the colon and keep it empty. It is therefore timely to examine the evidence which underpins this change in practice. At the same time, efforts have been made to standardise the nomenclature used to describe the clinical features of constipation. This will avoid the use of pejorative terms such “soiling” and facilitate meta-analyses.^1^^–^3

In many children, constipation is triggered by experience of painful bowel movements, caused by factors such as toilet training, changes in routine or diet, stressful events, intercurrent illness or delaying defaecation. The more time the faeces spend in the colon, the more water is reabsorbed. The stools then become harder and more difficult and painful to pass. Faecal impaction occurs when there is an accumulation of hard faeces in the rectum. Over time, the rectum distends, the urge to defaecate decreases, and there is a risk of faecal incontinence. After several days without a bowel movement, irritability, anorexia, abdominal distension and cramps may occur.^4^

Water makes up 75–80% of the weight of the normal stool, but a difference of only 10% in hydration will result in marked changes in stool consistency.^5^ PEG, a large molecular weight, water soluble polymer has the capacity to form hydrogen bonds with 100 molecules of water per molecule of PEG (MW 3350).^6^ When PEG is administered by mouth, the resulting hydration of the colonic content facilitates transit and painless defaecation in a linear dose-dependent fashion.^7^ Therefore PEG-based laxatives when used in escalating doses can also be used to completely remove faecal loading in preference to rectally-administered treatments.

Standard management of chronic constipation tends to begin with correction of dietary and lifestyle factors which predispose to the condition, in particular by increasing dietary fibre and fluid intake.^8^ However, dietary manipulation alone, including the use of corn syrup, was successful in resolving all symptoms of constipation in only 25% of children aged up to 2 years in one US study.^9^

Where simple measures fail, or where disimpaction is required, the next step involves one or more laxatives. An analysis of 13.5 million prescriptions for laxatives in England in 2005–6 shows that the most commonly chosen agents are the osmotic laxatives, accounting for 47% of prescriptions, stimulants (38%) and bulk-forming agents (15%).^10^

A comprehensive systematic review published in 2006 identified studies investigating treatments for childhood constipation and faecal impaction.^11^ Based on studies published up to June 2005, the authors reported that, despite very limited evidence, osmotic laxatives appeared to be the favoured option in childhood constipation, although the relative benefits of the various available treatments were unclear. They found no evidence to support the use of stimulant laxatives or bulk-forming agents in children.

Since 2005 several new randomised clinical trials investigating the efficacy of PEG have been published. The intention of this review, therefore, was to carry out a new literature search in order to ascertain whether more precise guidance can now be given with regard to osmotic laxatives and to further assess the evidence for their use in the treatment of constipation in children.

## Literature search

A primary search for randomised controlled trials was carried out in PubMed and EMBASE. The PubMed search used the following MeSH terms, which were mapped to appropriate EMTREE equivalents for the EMBASE search: Polyethylene glycols OR Lactulose OR Senna-Extract OR Bisacodyl OR {Picosulphate [Text word] OR Picosulfate [Text word]} AND Constipation OR Defecation OR Cathartics AND Infant OR Child, Preschool OR Child OR Adolescent AND Clinical Trial [Publication type]. The same search was repeated using Review [Publication type] in order to yield source material for a secondary reference search.

Additional electronic searches using similar strategies were carried out using the Cochrane Library and Google Scholar.

Reference lists of all identified clinical trials and reviews were scrutinised to identify further potentially relevant studies. Studies were examined to determine whether they complied with the inclusion criteria listed in box 1.

**Box 1 Inclusion and exclusion criteria for meta-analysis**

Inclusion

- Randomised trial of osmotic laxative versus
-
  - placebo
  - active comparator
- Participants aged <18 years
- Diagnosis of constipation of >3 months’ duration in the absence of structural, endocrine or metabolic disease
- Quantitative effect on constipation recorded as outcome

Exclusion

- Retrospective analyses
- Non-comparative studies
- Constipation secondary to other diagnoses
- Not been published in a peer-reviewed journal

Qualitative appraisal for potential bias according to the following aspects of design

- Inclusion criteria
- Quality of randomisation
- Presence and quality of blinding
- Washout in crossover studies
- Handling of withdrawals and dropouts

Data analysis

- Lack of consistency in outcomes
- Variation in design
- Recruitment strategy
- Diagnostic criteria
- Treatment protocols

NB. Studies precluded from inclusion by data analysis are presented narratively.

## RESULTS

### Literature search

**Figure 1:**
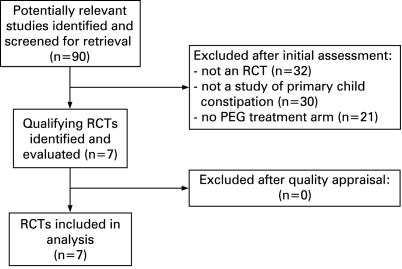
Literature search flow chart. PEG, polyethylene glycol; RCT, randomised controlled trial.

The initial PubMed search yielded 55 possibly relevant clinical studies and 12 review articles. The EMBASE search yielded 45 possibly relevant clinical studies and 59 review articles. On examination of full-text copies of the research papers, seven were found to meet the inclusion criteria^12^^–^18 while the rest were rejected (fig 1).

The subsidiary search of other databases and the reference list review yielded no additional qualifying studies.

The seven separate relevant studies that were identified for consideration in the review included data on 594 children treated for constipation (table 1). Study types were as follows:

**Table 1:** Summary of included studies

Study	Study duration	Treatment	Dosage	Total n	Success	Defaecation rate
Thomson *et al*^12^	2-Week crossover	PEG 3350+E	Mean 0.6–0.7 g/kg/day	51	83%	3.12 per week
		Placebo	N/A	51	21%	1.45 per week, p<0.001
Candy *et al*^13^	12 Weeks	PEG 3350+E	Mean 11.8 g/day	28	–	9.4 per week
		Lactulose	Mean 24.1 g/day	30	–	7.9 per week, p = 0.007
Dupont *et al*^14^	12 Weeks	PEG 4000	4–8 g/day±enemas	51	–	8.5/7 per week
		Lactulose	3.33–6.66 g/day±enemas	45	–	11.5/6 per week, p = NS (results: toddlers/babies)
Voskuijl *et al*^15^	8 Weeks	PEG 3350	Mean dose 5.4 g/day	50	56%	7.1 per week
		Lactulose	Mean dose 6.7 g/day	50	26%, p = 0.02	6.4 per week, p<0.01
Gremse *et al*^16^	2-Week crossover	PEG 3350	0.3 g/kg/day	44	84%	14.8 per 2 weeks
		Lactulose	1.3 g/kg/day	44	46%, p = 0.002	13.5 per 2 weeks
Wang *et al*^17^	2 Weeks	PEG 4000	20 g/day	105	70%	7 per week (median)
		Lactulose	10 g/day	111	40%, p<0.05	6 per week, p<0.05
Loening-Baucke *et al*^18^	12 Months	PEG 3350	Mean 0.6 g/kg/day	39	62%	6.8 per week
		Milk of magnesia	Mean 1.2 ml/kg/day	40	43%, p = NS	8.2 per week, p = NS

E, electrolytes; NS, not significant; PEG, polyethylene glycol.

- one randomised, controlled trial of PEG versus placebo^12^
- five randomised, controlled trials of PEG versus lactulose^13^^–^17
- one randomised, controlled trial of PEG versus milk of magnesia.^18^

Differences in study design, patient populations and treatment protocols meant that meta-analysis could not be carried out in a meaningful fashion. The results of the studies, together with quality appraisals, are therefore detailed individually.

### Treatment outcomes

#### PEG versus placebo

A total of 51 children were included in this randomised double-blind crossover study that was carried out in six paediatric outpatient centres.^12^ All were aged between 2 and 11 years and had at least a 3-month history of ⩽2 complete defaecations per week combined with one or more of the following: pain on defaecation on ⩾25% of days, ⩾25% of bowel movements with straining or ⩾25% of movements with hard/lumpy stools. Patients with current or previous faecal impaction were excluded, as were those taking high doses of stimulant laxatives with no effect.

Patients were randomised to receive either PEG 3350+electrolytes (PEG+E) for 2 weeks, followed by placebo, after a 2-week washout period, or the reverse. Dose was titrated according to age and response and ranged from one to nine 6.9 g PEG+E sachets daily. Randomisation was by computer allocation and blinding was maintained using a double dummy approach. The primary outcome measure was the number of complete defaecations per week.

At the end of 6 weeks, four children failed to provide sufficient bowel movement data for inclusion. Based on the remaining 47 participants, mean PEG dosage used was 0.6 g/kg in 2–6-year-old children and 0.7 g/kg in 7–11-year-old children. There was a significant difference in the number of complete defaecations: 3.12 vs 1.45, mean difference 1.64, p<0.001 and all defaecations 5.68 vs 4.10, mean difference 1.58, p = 0.003.

The authors also provided a per protocol analysis, which was restricted to those with at least 7 days of data from each treatment period and no concomitant laxative use. For this subgroup of 36 patients, the benefit was somewhat enhanced: mean defaecations 3.63 vs 1.63, mean difference 1.96, p<0.001.

#### PEG versus lactulose (five studies)

In the first study,^13^ 63 children with faecal impaction were recruited for an open label evaluation of PEG+E for the treatment of faecal impaction at a single paediatric centre. On completion of this phase of the study, 58 progressed to randomisation with lactulose or PEG+E as maintenance therapy. Of the five who were not randomised, two withdrew from the study and three failed to disimpact within 7 days. All those entering the study were between 2 and 11 years of age and had at least a 3-month history of constipation which had failed to be controlled during normal outpatient management.

A randomisation list was supplied by a third party (the study sponsor) in order to maintain concealment of allocation from the investigator and both medications were supplied as dry powders for the patient to reconstitute. Treatment was titrated by response and was not dictated by protocol. If required, patients were also allowed to use supplemental senna. The primary outcome measure was the number of successful defaecations per week on treatment, evaluated after 12 weeks.

The mean daily dose of PEG was 11.8 g and the mean dose of lactulose was 24.1 g. Overall, 31% of lactulose patients required senna, while no PEG+E patients needed senna. Of the 58 children who started, 10 failed to complete the study. Seven patients taking lactulose reimpacted and were therefore withdrawn, compared with none taking PEG+E. Two further lactulose patients withdrew from the study, while one PEG patient failed to complete a diary card. On-treatment efficacy data were available for 53 patients, which provided the basis for the primary intention to treat analysis: the mean number of successful defaecations per week was significantly greater in children given PEG+E than in those on lactulose: 9.4 vs 5.9, mean difference 3.5, p = 0.007.

The second study^14^ investigated PEG 4000 versus lactulose. Ninety six children were included in this randomised double-blind parallel group study that was carried out in 30 paediatric centres. Twenty two children were aged between 6 and 12 months and had at least a 1-month history of ⩽1 stool per day. The remaining 74 were aged 1–3 years and had at least a 3-month history of ⩽3 stools per week.

The randomisation strategy was not described. Medications were supplied as dry powders for the patient to reconstitute and a double-dummy approach employed in order to conceal treatment allocation. In patients under 1 year of age, treatment was limited to 4 g PEG or 3.33 g lactulose. In older children this could be doubled, if required, with glycerol microenemas being used if this still proved inadequate. No other laxatives were permitted. The primary outcome of this study was a biochemical assessment of safety after 6 and 12 weeks of treatment, although stool frequency was also evaluated as a secondary outcome.

Eight children failed to complete the study (four in each group), although only one (in the lactulose group) dropped out due to treatment failure. The median daily dose of PEG was 4 g in both babies and older children. For lactulose, the dose was 3.33 g in babies and 3.66 g in older children. Thirty per cent of children in the PEG group required enemas in the first half of the study and 17% in the second half versus 43% and 41% in the lactulose group (p = 0.012). At 6 weeks, the median numbers of stools per week in the PEG and lactulose groups, respectively, were 8.5 vs 11, p = NS (babies) and 8 vs 6, p<0.013 (toddlers). At 12 weeks, the equivalent results were 8.5 vs 11.5, p = NS (babies) and 7 vs 6, p = NS (toddlers).

The third study^15^ was a multicentre double-blind parallel group study carried out in three hospitals, comparing PEG 3350 (without electrolytes) with lactulose in 100 children. Participants were aged 6 months to 15 years and had to have experienced two or more of the following for the preceding 3 months: <3 bowel movements/week, >1 faecal incontinence/week, large amount of stool every 7–30 days, palpable abdominal/rectal mass.

Patients were randomised to receive PEG or lactulose at a starting dose of 2.95 g or 6 g, respectively, for those aged under 6 years of age and 5.9 g or 12 g for those aged over 6 years of age. The randomisation method was not described. Medications were dispensed as dry powder in identical unlabelled sachets with investigators and patients blinded to assignation. Doses were titrated according to response, with the option to add in bisacodyl if required. The primary outcomes were stool frequency, faecal incontinence and overall treatment success at 8 weeks.

Ninety one of the original 100 participants completed the trial. Two patients in each group were lost to follow-up. One patient withdrew from the PEG group due to dislike of the taste. Two patients were withdrawn from the lactulose group as they were found to be Helicobacter positive. The reason for withdrawal for one patient in each group was not stated. At 8 weeks the mean dose of PEG was 5.4 g/day and lactulose 13.9 g/day. Bisacodyl was used by nine patients in the PEG group and 10 in the lactulose group. Per protocol analysis showed a mean weekly stool frequency at 8 weeks in the PEG and lactulose groups, respectively, of 7.12 and 6.43 (p = NS). In both groups there was significant improvement versus baseline. There was also no significant difference in the frequency of faecal incontinence. However, the rate of overall treatment success (stool ⩾3/week+incontinence <1/fortnight) was significantly better in PEG treated patients than in those on lactulose (56% vs 29%, p = 0.02).

The fourth PEG-lactulose study^16^ compared the use of PEG 3350 (without electrolytes) with lactulose in a randomised crossover single centre design. Forty four children aged 2–16 years were recruited. All had been referred to a specialist unit for treatment of their constipation, but no explicit diagnostic criteria are listed. Mean baseline stool frequency was 1.7 per week. Patients were randomised to receive 10 g/m^2^/day PEG or 1.3 g/kg/day lactulose at a fixed dose for 2 weeks. The randomisation strategy is not detailed and treatment was not blinded. Various outcomes were recorded, including stool frequency, but it is unclear whether this was the primary outcome.

Of the 44 patients who started the study, seven withdrew due to lack of efficacy (six in the lactulose group and one in the PEG group). No intention to treat analysis is given. A per-protocol analysis, comparing the mean number of bowel movements over 14 days, is presented for the remaining 37 patients: 14.8 vs 13.5 for PEG and lactulose, respectively. Based on global assessment by the parent or guardian, 84% of the PEG-treated patients achieved a satisfactory outcome compared with 46% of the lactulose-treated patients (p<0.002).

The final study^17^ was a multicentre open label parallel group study carried out in seven hospitals in China, comparing PEG 4000 with lactulose in 216 children. Participants were aged 8–18 years (mean 11.2 years) and had a minimum 3-month history of constipation, with a baseline Bristol Stool Form Scale of less than 3.

Patients were randomised to receive PEG or lactulose at a fixed dose of 20 g (PEG) or 10 g (lactulose) daily for 2 weeks. Doses could be reduced according to clinical response, if required. The randomisation method is not known and treatment was not blinded. Outcomes assessed included stool frequency, stool consistency and complete constipation remission rate.

All the participants completed the 14-day trial. Intention to treat analysis showed an increase in median weekly stool frequency in both groups. In PEG-treated patients the median increased from 2/week at baseline to 7/week; in lactulose-treated patients the median increased from 2/week at baseline to 6/week. The between-treatments difference was statistically significant. PEG was also significantly better in terms of the proportions with complete resolution of constipation (72% vs 41%, p<0.05), mean Bristol Stool Form Scale (4.26 vs 3.63, p<0.05) and resolution of abdominal pain (75% vs 57%, p<0.05).

#### Brief summary of comparisons versus other laxatives (one study)

One study^18^ has compared PEG 3550 with milk of magnesia in a randomised open label parallel group study of 79 children aged 4–16 years that lasted 12 months. There was no significant difference between groups in either mean number of bowel movements or episodes of faecal incontinence over the course of the study. However, more children refused treatment with milk of magnesia than with PEG (35% vs 5%, p<0.001).

## DISCUSSION

Previously published reviews on the management of childhood constipation have commented on the lack of high quality clinical trials in the field,^11^ ^19^^–^21 despite the widespread use of laxatives. Although there remain significant gaps in the evidence base, this literature review has demonstrated that the evidence base of current clinical practice is now improving.

The placebo-controlled study of PEG+E by Thomson *et al*^12^ defines a baseline proof of principle in a relatively mildly affected population, while the two phase study by Candy *et al*^13^ confirms that, once disimpacted, even severely affected children can be maintained satisfactorily on low doses of PEG+E (the mean daily dose was just under two paediatric dose sachets). PEG+E could also be used as monotherapy whereas almost one third of patients on lactulose required senna. No patient on PEG+E reimpacted compared with almost one in four on lactulose.

The five studies comparing PEG with lactulose^13^^–^16 exhibit some potentially important differences that prevent the results being pooled in meta-analysis. Firstly, they involve three different agents: PEG 3350, PEG 3350+E and PEG 4000. While there is no a priori reason for supposing that these will behave functionally differently as laxatives, one cannot necessarily assume equivalence. Secondly, there are dose differences: in two cases^13^ ^15^ the dose was titrated according to clinical response, subject to an upper limit; in one case^14^ a single titration was permitted, with add-in therapy being required beyond that; and in the other two studies^16^ ^17^ there was a fixed dose regime. Finally, there are methodological concerns regarding the analysis of the Gremse *et al* study^16^ in that the presented results exclude the patients who had withdrawn due to lack of efficacy, biasing the results in favour of lactulose.

There have been no clinical studies which have compared PEG with PEG+E. Of the seven studies reviewed, five compared PEG and two compared PEG+E with either placebo or another laxative.

Despite these issues, there appears to be a consensus amongst the studies that treatment with PEG is more effective than with lactulose, with neither agent appearing to have a convincing advantage in terms of tolerability. PEG-based laxatives confer the advantage that in high doses they can be used for disimpaction, and, as they are not fermented by colonic bacteria, do not result in increased gas production.

The comparison of PEG versus milk of magnesia was a well designed and executed study relating to an agent that would not be widely regarded as a first line agent in Europe. Nonetheless, it adds to our understanding of these agents both in terms of efficacy and tolerability.

## CONCLUSIONS

Chronic constipation is associated with long term problems including megarectum, reduced sensitivity of the rectum to the presence of faeces, and abnormal gut motility. Effective treatment of constipation has been shown to encourage the rectum to revert to normal size.^22^ Treatment has also been shown to improve rectal sensitivity in patients who responded to treatment, if not in those who were non-responders,^23^ and can improve gut motility in children for up to 3 years.^24^ Nevertheless, even after treatment, chronic constipation may have long term sequelae. Abnormal ano-rectal functions may persist for years after cessation of treatment and recovery,^25^ while constipation may continue to be an intermittent problem in children with faecal impaction in whom gut motility has returned to normal after treatment.^24^ Managing chronic constipation in children effectively and early in its course may therefore be important in preventing long term defaecation disorders. In order to achieve this it is essential that treatment regimes are critically reviewed in the light of emerging evidence.
